# SARC018_SPORE02: Phase II Study of Mocetinostat Administered with Gemcitabine for Patients with Metastatic Leiomyosarcoma with Progression or Relapse following Prior Treatment with Gemcitabine-Containing Therapy

**DOI:** 10.1155/2018/2068517

**Published:** 2018-10-24

**Authors:** Edwin Choy, Karla Ballman, James Chen, Mark A. Dickson, Rashmi Chugh, Suzanne George, Scott Okuno, Raphael Pollock, Rajiv M. Patel, Antje Hoering, Shreyaskumar Patel

**Affiliations:** ^1^Massachusetts General Hospital, Division of Hematology Oncology, 55 Fruit Street, Boston, MA 02114, USA; ^2^Weill Cornell Medicine Healthcare Policy and Research, 402 East 67th Street, LA-225, New York, NY 10065, USA; ^3^The Ohio State University Comprehensive Cancer Center, 1800 Cannon Drive, 250 Lincoln Tower, Columbus, OH 43210, USA; ^4^Memorial Sloan Kettering Cancer Center and Weill Cornell Medical College, 300 E. 66th Street, New York, NY 10065, USA; ^5^University of Michigan Comprehensive Cancer Center, 1500 E. Medical Center Drive, Rm C-407 MIB/SPC 5843, Ann Arbor, MI 48109, USA; ^6^Dana-Farber Cancer Institute, Center for Sarcoma and Bone Oncology, 450 Brookline Ave, D-1212, Brookline, MA 02215, USA; ^7^Mayo Clinic, 200 First St. SW, Rochester, MN 55905, USA; ^8^Ohio State University Wexner Medical Center, 410 West 10th Avenue, N924 Doan Hall, Columbus, OH 43206, USA; ^9^Michigan Medicine, Department of Pathology, 1301 Catherine Street, SPC 5602, 3261G Medical Science I, Ann Arbor, MI 48109-5602, USA; ^10^Cancer Research and Biostatistics, 1730 Minor Ave, Suite 1900, Seattle, WA 98101, USA; ^11^MD Anderson Cancer Center, Sarcoma Medical Oncology, 1400 Holcombe Blvd, Unit 450, Houston, TX 77030, USA

## Abstract

Histone deacetylase inhibitors (HDACi) can reverse chemoresistance, enhance chemotherapy-induced cytotoxicity, and reduce sarcoma proliferation in cell lines and animal models. We sought to determine the safety and toxicity of mocetinostat and its ability to reverse chemoresistance when administered with gemcitabine in patients with metastatic leiomyosarcoma resistant to prior gemcitabine-containing therapy. Participants with metastatic leiomyosarcoma received mocetinostat orally, 70 mg per day, three days per week, increasing to 90 mg after three weeks if well tolerated. Gemcitabine was administered at 1,000 mg/m^2^ intravenously at 10 mg/m^2^/minute on days five and 12 of every 21-day cycle. Disease response was evaluated with CT or MRI. Twenty participants with leiomyosarcoma were evaluated for toxicity. Median time to disease progression was 2.0 months (95% CI 1.54–3.12). Eighteen participants were evaluated for radiologic response by RECIST 1.1. Best responses included one PR and 12 SD. Tumor size reduced in 3 patients. Most common toxicities were fatigue, thrombocytopenia, anemia, nausea, and anorexia. One patient experienced a significant pericardial adverse event. No study-related deaths were observed. Rechallenging with gemcitabine by adding mocetinostat was feasible and demonstrated modest activity in patients with leiomyosarcoma. Further studies are needed to better define the role of HDAC inhibitors in patients with metastatic leiomyosarcoma.

## 1. Introduction

Leiomyosarcoma is a relatively common histologic subtype of soft tissue sarcoma that is usually incurable after development of metastasis [1]. Although cytotoxic chemotherapies such as doxorubicin [2–4], gemcitabine [5–7], and docetaxel [5, 8] can provide temporary benefit in some patients with metastatic leiomyosarcoma, these agents have modest clinical effectiveness [9, 10]. While gemcitabine does have single agent activity in leiomyosarcoma, combining docetaxel with gemcitabine yielded improvements in response rates, as well as progression-free and overall survival [11–15]. The most recent agents approved by the FDA for treatment of this disease include the multityrosine kinase inhibitor, pazopanib (Votrient), and a DNA binder, trabectedin (Yondelis) [16–18]. Neither of these drugs was shown to improve overall survival [19, 20]. Therefore, more effective treatments are needed.

We were interested in the role histone acetylation/deacetylation plays as a potential treatment strategy for sarcomas that are typically insensitive to traditional chemotherapeutic agents. Transcriptionally active genes are associated with hyperacetylated chromatin, while transcriptionally silent genes are associated with hypoacetylated chromatin [21, 22]. Chromatin acetylation is controlled by the opposite effects of two families of enzymes: histone acetyltransferases (HATs) and histone deacetylases (HDACs). HATs, as transcription coactivators, catalyze the addition of acetyl groups on the amino group of lysine residues in the *N*-terminal tails of core histones. Conversely, HDACs, as transcription corepressors, remove the acetyl groups from the acetylated lysines in histones [23]. Deregulation of HDAC activity can cause malignant diseases in humans [24].

Small molecule inhibitors of HDACs have emerged as a therapeutic class of molecules with anticancer potential [25, 26]. Anticancer activity of HDACi is mediated by regulating aberrant gene expression at the transcriptional level in cancer cells. These gene expression changes lead to inhibition of proliferation, induction of apoptosis, and/or cell differentiation in cancer cells *in vitro* and *in vivo*. Inhibitors of histone deacetylase (HDACi) have demonstrated preclinical activity in sarcoma models [27–36], and there have been anecdotes of patients with sarcoma responding to HDACi therapy [37]. There has been recent interest in using HDACi as synergistic therapy with chemotherapy for sarcomas [28,38–46]. We have recently completed a phase I study of an HDACi with chemotherapy in patients with metastatic sarcoma [47].

Histone deacetylase inhibitors have been observed to enhance apoptosis of cancer cells induced by several chemotherapeutic agents including gemcitabine [43,48–53]. Mocetinostat (MGCD0103) is an orally bio-available drug that has significant antitumor activity *in vivo* against a broad spectrum of human cancer types, and antitumor activity is achieved at clinically achievable doses [54–56]. Mocetinostat interacts synergistically with gemcitabine to inhibit cancer cell growth *in vitro* and *in vivo* [54, 56, 57]. These results suggest that a combination regimen with the HDAC inhibitor mocetinostat and gemcitabine may be a valuable therapeutic strategy to reverse chemoresistance in patients with gemcitabine-resistant leiomyosarcoma.

## 2. Materials and Methods

The study was an open label multicenter Phase II trial conducted as a part of the SARC (Sarcoma Alliance for Research through Collaboration) SPORE grant (U54CA168512) and registered on clinicaltrials.gov under the NLM identifier NCT02303262. The study protocol and consent forms were reviewed and approved by each of the participating institutions' institutional review boards. All patients participated in informed consent procedures prior to screening for eligibility. The patient group was comprised of adult patients who were diagnosed with leiomyosarcoma. As all patients were enrolled at academic centers of sarcoma excellence participating in the SPORE project, no central re-review of pathology was required. These patients had previously demonstrated disease progression by RECIST 1.1 either while receiving gemcitabine or within six months after completing a course of chemotherapy using a gemcitabine-based regimen. No limits on numbers of prior therapy were required for study entry.

After participants were confirmed eligible to participate in the study, each received 70 mg mocetinostat (provided by Mirati Therapeutics, Inc.) per day for three days per week in combination with gemcitabine, administered at 1000 mg/m^2^ at a rate of 10 mg/m^2^/minute [58–60] on days five and 12 of each 21-day cycle. The dose for mocetinostat was escalated to 90 mg/dose starting with cycle two if no grade three or four clinically significant toxicities or any new pericardial effusions were observed during the first cycle.

Because pericardial adverse events have been reported with mocetinostat treatment, participants underwent ECG screening on days one, five, and 12 of the first cycle, and cardiac ultrasound at screening, day 12 of cycles one and two, and before each subsequent cycle of therapy.

Study participants underwent CT imaging of the chest, abdomen, and pelvis prior to beginning cycle one and then every two cycles until disease progression; at which point, they were removed from study treatment.

All patients (including those who discontinued early) were followed for adverse events from enrollment into the study to at least 30 days after removal from the study or until death. The participants who were removed from study for unacceptable adverse events were followed until resolution or stabilization of the adverse event.

### 2.1. Statistical Design

This study was designed as a two-stage phase II clinical trial. Analysis was planned to be performed after 20 patients were enrolled (completion of stage I) to determine whether an additional 20 patients (stage II) should be enrolled. The primary objective of the study is to determine the rate of tumor response to treatment with mocetinostat and gemcitabine. Response to therapy was assessed by CT or MRI using RECIST 1.1 criteria. Response rates (CR or PR) were calculated as the number of patients achieving a response divided by the number of patients having been evaluated for response. 95% confidence intervals (CI) based on the binomial distribution were calculated. The Kaplan–Meier estimator was used to summarize the progression-free survival (PFS).

We would have considered combination therapy with mocetinostat and gemcitabine to be worthy of further evaluation if the true response rate was >20%, with the null hypothesis being that a <5% response rate would be seen with an ineffective treatment. Assuming that the number of successes is binomially distributed, this study had a one-sided alpha of 0.05 and a power of 0.92 for detecting a true clinical benefit rate of at least 20% versus the null hypothesis of 5% or less. This translated to a decision rule that the second stage of patients would be accrued if one or more responses (CR or PR) were seen in the first 20 patients. The treatment regimen would have been declared worthy of further study if five or more out of 40 patients had a response.

All patients who had initiated treatment were considered evaluable for adverse event analysis. The maximum grade of each adverse event was recorded for each patient and frequency tables for each adverse event observed were generated.

## 3. Results

A total of 20 patients (all with prior tumor growth after gemcitabine-containing therapy) were enrolled across five participating sites during the first stage. There were 18 patients evaluable for radiologic response. Eight of these patients had uterine leiomyosarcoma. Two patients withdrew consent prior to being evaluated for response. Reasons for withdrawal were to initiate hospice care and to avoid further adverse events the patients were experiencing.

Table 1 displays patient demographics and other characteristics at baseline. The median age was 57.5 years old (range: 39–71 years old). All patients had metastatic sarcoma at the time of enrollment in the trial and had been previously treated with a gemcitabine-containing regimen.

All patients received at least one dose of mocetinostat and were evaluable for adverse events. A summary of adverse events (AEs), regardless of treatment attribution, is given in Table 2. Overall, 18 (90%) patients experienced a grade three or four AE: 11 (55%) patients experienced a nonhematological and 14 (70%) experienced a hematological grade three or four AEs. The most common AEs were fatigue, thrombocytopenia, anemia, and neutropenia. No study-related deaths were observed. Six patients had trace to minimal pericardial effusions. One patient had a small pericardial effusion, which was grade two. One patient experienced grade three pericardial adverse events. This was a 46-year-old woman who was hospitalized after grade three pericardial effusion which was seen on cardiac ultrasound on cycle one, day 12. This resulted in pericarditis, early cardiac tamponade, and pleural effusions. A summary of significant cardiac ultrasound findings is given in Table 3.

**Figure 1:**
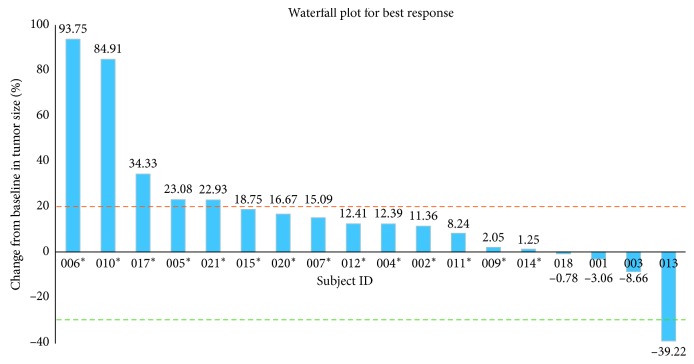
Waterfall plot for best response. The subject IDs with “^*∗*^” are those with progressive disease in the study. “PR” means partial response. The dotted lines indicate a 30% reduction and 20% increase from baseline, which are the cutoff points that determine partial response and progressive disease, respectively. The value for each vertical bar is added.

**Figure 2:**
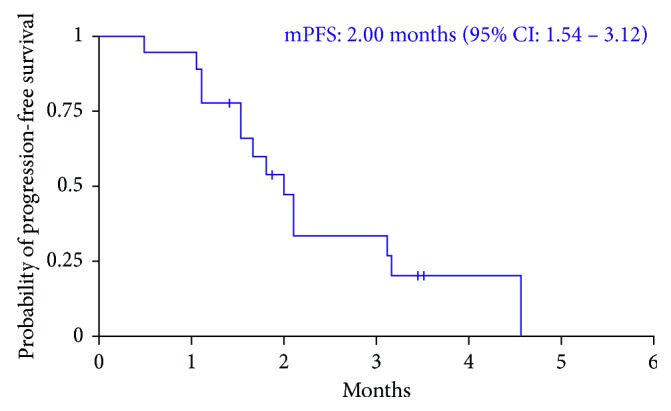
The Kaplan–Meier curve for progression-free survival.

In the 18 participants evaluable for radiologic response, there was one PR (seen in a patient with uterine leiomyosarcoma), five PD (two with uterine leiomyosarcoma, two with leiomyosarcoma in the peritoneum, and one in the retroperitoneum), and 12 SD (five of these patients had uterine leiomyosarcoma, two had renal leiomyosarcoma, and five with others) as best response (Figure 1), with two participants completing five cycles or more. The median progression-free survival was two months (95% CI: 1.5 to 3.1) (Figure 2). In the one participant who did experience a PR, her target lesion was a single lung nodule. When a study-required postdrug biopsy was attempted, she experienced a clinically significant pulmonary bleed that required ICU support. Although she eventually recovered from this episode, another lung biopsy was not attempted due to safety concerns. Therefore, pathologic evaluation to confirm metastases and evaluate pharmacodynamic parameters was not possible. Given this uncertainty and the limited clinical benefit, if any, that was observed for this patient, SARC and Mirati Therapeutics, Inc. determined that the modest response rate observed at interim analysis did not justify further enrollment, and the study was halted after completion of the first stage.

## 4. Discussion

Numerous clinical trials have been performed to identify clinical activity of HDAC inhibitors in patients with solid tumors. Although HDAC inhibitors have shown success in preventing graft versus host disease (GVHD) [61] and treating patients with T-cell lymphoma [62] and multiple myeloma [63, 64]; to date, no study has shown significant benefit in patients with solid tumors [65, 66]. We sought to explore the potential for HDAC inhibition to reverse chemoresistance based on promising results seen in animal models of leiomyosarcoma. We chose mocetinostat as our model HDAC inhibitor because prior preclinical studies have shown synergy with gemcitabine [52] as measured by *in vitro* growth inhibition and apoptosis of PANC1 and BxPC3 pancreatic cancer cells. In a phase I/II study of mocetinostat and gemcitabine in patients with refractory solid tumors [67], the maximum tolerated dose of mocetinostat was 90 mg per dose, three doses per week, when administered with 1000 mg/m^2^ gemcitabine, given weekly for three weeks in every 28-day cycles. DLTs included fatigue, abdominal pain, deep vein thrombosis, diarrhea, nausea, mental status change, thrombocytopenia, and vomiting. The phase II portion of this study focused on patients with pancreatic cancer.

This dose and schedule of administration for our current study was modified from this previously conducted phase I/II study. The timing of administration of gemcitabine was modified to days 5 and 12 in order to ensure patients were predosed sufficiently with mocetinostat to allow for concurrent drug exposure to tumor cells. The previously published study administered gemcitabine on day 1 of each week. Given the short half-life of gemcitabine, this would not have allowed for prolonged coexposure of tumor cells to both agents simultaneously. Additionally, the prior study reported that 81% of all patients experienced grade 3 or greater treatment-related adverse events. Therefore, in designing the current study, we opted to start with a lower dose of mocetinostat at 70 mg for one cycle to evaluate tolerability before advancing to the MTD/RP2D dose of 90 mg reported in the prior study.

To show the relative benefits of adding mocetinostat to gemcitabine therapy would involve a large randomized clinical trial that would have been logistically challenging in sarcoma. Therefore, we initially focused our study on the selected patients who already demonstrated chemoresistance to gemcitabine. In such a cohort of patients, even a small number of tumor responses to therapy would be a significant proof of principle that mocetinostat could reverse chemoresistance as the null hypothesis is that no responses would be seen if mocetinostat was an inactive drug. This allowed us to test a relatively modest-size cohort for the proof of concept that mocetinostat would reverse gemcitabine chemoresistance.

We enrolled only patients with metastatic leiomyosarcoma who progressed either during treatment with gemcitabine or within six months of completing treatment with gemcitabine. The adverse events that we observed were largely expected and observed in prior clinical trials using mocetinostat. Pericardial SAEs, for example, were seen in 19 cases (4.3%) of the 435 patients who had previously received mocetinostat prior to this study. Based on this finding, we incorporated frequent cardiac ultrasound monitoring to be performed at screening, during cycle one, and at every cycle of the study. The current study observed six cases of trace to minimal and clinically insignificant pericardial effusion, one case of grade 2 pericardial effusion (listed in Table 3), and one case of significant grade 3 pericardial effusion that led to pericarditis and cardiac tamponade (listed in Table 3).

Overall, in the context that the subjects had metastatic disease that would be universally fatal unless an effective treatment can be identified, we found that the combination of mocetinostat and gemcitabine was relatively well tolerated. However, the median progression-free survival for participants was short, and we did not observe significant response rates as determined by RECIST.

## 5. Conclusions

Although we showed that mocetinostat can be safely combined with gemcitabine in this study population, our study did not demonstrate that mocetinostat can reverse chemoresistance in patients with previously established gemcitabine-resistant leiomyosarcoma. However, we studied only patients with metastatic leiomyosarcoma who had previously progressed on a gemcitabine-containing chemotherapy regimen. As these patients were selected for their chemoresistant tumor characteristics, our study observations do not negate a potential role for HDAC inhibitors as a synergistic mechanism in chemotherapy naïve patients or for patients with other solid tumors. As several minor responses were seen in this study, additional studies are needed to better define a role for HDAC inhibitors in patients with sarcoma.

## Figures and Tables

**Table 1:** Patient characteristics (total number of patients = 20).

Variable		*N* (%)
*Site*		
Dana-Farber Cancer Institute		3 (15)
Massachusetts General Hospital		3 (15)
Memorial Sloan Kettering Cancer Center		5 (25)
Ohio State University		6 (30)
University of Michigan		3 (15)

*Sex*		
Female		14 (70)
Male		6 (30)

*Ethnicity*		
Hispanic or Latino		1 (5)
Not Hispanic or Latino		19 (95)

*Race*		
Asian		2 (10)
Black or African heritage		3 (15)
White		15 (75)

*Tumor location at diagnosis*		
Kidney		2 (10)
Liver		1 (5)
Others		6 (30)
Pelvis		1 (5)
Peritoneum		2 (10)
Uterus		8 (40)

*Metastasis present at diagnosis*		
No		16 (80)
Yes		4 (20)

*Site of metastasis*		
Abdomen		1 (5)
Colon		2 (10)
Kidney		1 (5)
Liver		4 (20)

Lung		12 (60)
Pancreas		1 (5)
Pelvis		2 (10)
Peritoneum		1 (5)
Spine		3 (15)
Thyroid		1 (5)
Others		3 (15)
More than one site of metastasis		6 (30)

*Received prior radiation therapy*	
No		14 (70)
Yes		6 (30)

*Received prior surgery*	
No		3 (15)
Yes		17 (85)

*Prior regimens*	
Gemcitabine/docetaxel		20 (100)
Gemcitabine/vinorelbine		1 (5)
AIM (doxorubicin, ifosfamide, and mesna)		3 (15)
Dacarbazine		5 (25)
Doxorubicin		9 (45)
Ifosfamide		1 (5)
Pazopanib		7 (35)
Trabectedin		1 (5)
Others		10 (50)
*Number of prior lines of therapy before study entry*	
One		6
Two		3
Three		2
Four		4
Five		2
Six		1
Eight		2

**Table 2:** All adverse events regardless of attribution (*N* = 20 patients).

Adverse events, *n* (%)	Grade 1	Grade 2	Grade 3	Grade 4
Anemia	1 (5)	4 (20)	5 (25)	1 (5)
Fatigue	5 (25)	5 (25)	3 (15)	
Neutropenia			6 (30)	3 (15)
Thrombocytopenia			3 (15)	2 (10)
Peripheral sensory neuropathy	1 (5)		2 (10)	
Lymphopenia		1 (5)	2 (10)	
Diarrhea	3 (15)		1 (5)	
Decreased ejection fraction			1 (5)	
Hypokalemia			1 (5)	
Pneumonia			1 (5)	
Noncardiac chest pain			1 (5)	
Pain			1 (5)	
Pericardial effusion^*∗*^			1 (5)	
Pericardial tamponade^*∗*^			1 (5)	
Pericarditis^*∗*^			1 (5)	
Pulmonary embolism			1 (5)	
Syncope			1 (5)	
Vomiting	4 (20)		1 (5)	
Leukopenia		1 (5)	2 (10)	
Biopsy-related bleeding				1 (5)

^*∗*^Grade 3 pericardial effusion, tamponade, and pericarditis all occurred in the same patient.

**Table 3:** Significant cardiac ultrasound findings.

Patient	Ejection fraction at baseline (%)	Ejection fraction range (%)	Notable changes
1	61	57–63	
2	59	58–64	C1D1: there was mild eccentric left ventricular hypertrophy. There is mild diastolic dysfunction (impaired relaxation pattern with normal filling pressure) C2D1: there was mild diastolic dysfunction (impaired relaxation pattern with normal filling pressure)
3	66	55–66	
4	65	55–65	
5	65	60–65	
6	64	60–64	C1D12: grade 3 pericardial effusion, pericarditis, and tamponade
7	76	76–81	
8	76	67–76	C1D12: mild eccentric left ventricular hypertrophy (increased left ventricular mass with normal relative wall thickness) C2D1: (1) small left ventricular cavity size suggestive of an underfilled left ventricle; (2) there is mild diastolic dysfunction (impaired relaxation pattern with normal filling pressures)
9	55	50–59	
10	69	69–73	
11	55	55–65	
12	68	68–74	
13	55	38–55	C3D1: grade 3 reduction in cardiac ejection fraction
14	67	66–69	C4D1: grade 2 pericardial effusion
15	58	55–60	
16	63	60–63	
17	59	59–60	
18	55	55–65	
19	64	61–69	
20	60	60–60	

## Data Availability

The data used to support the findings of this study are available from the corresponding author upon request.
