# Corrigendum to “SARC018_SPORE02: Phase II Study of Mocetinostat Administered with Gemcitabine for Patients with Metastatic Leiomyosarcoma with Progression or Relapse following Prior Treatment with Gemcitabine-Containing Therapy”

**DOI:** 10.1155/2019/7608743

**Published:** 2019-08-27

**Authors:** Edwin Choy, Karla Ballman, James Chen, Mark A. Dickson, Rashmi Chugh, Suzanne George, Scott Okuno, Raphael Pollock, Rajiv M. Patel, Antje Hoering, Shreyaskumar Patel

**Affiliations:** ^1^Massachusetts General Hospital, Division of Hematology Oncology, 55 Fruit Street, Boston, MA 02114, USA; ^2^Weill Cornell Medicine Healthcare Policy and Research, 402 East 67th Street, LA-225, New York, NY 10065, USA; ^3^The Ohio State University Comprehensive Cancer Center, 1800 Cannon Drive, 250 Lincoln Tower, Columbus, OH 43210, USA; ^4^Memorial Sloan Kettering Cancer Center and Weill Cornell Medical College, 300 E. 66th Street, New York, NY 10065, USA; ^5^University of Michigan Comprehensive Cancer Center, 1500 E. Medical Center Drive, Rm C-407 MIB/SPC 5843, Ann Arbor, MI 48109, USA; ^6^Dana-Farber Cancer Institute, Center for Sarcoma and Bone Oncology, 450 Brookline Ave, D-1212, Brookline, MA 02215, USA; ^7^Mayo Clinic, 200 First St. SW, Rochester, MN 55905, USA; ^8^Ohio State University Wexner Medical Center, 410 West 10th Avenue, N924 Doan Hall, Columbus, OH 43206, USA; ^9^Michigan Medicine, Department of Pathology, 1301 Catherine Street, SPC 5602, 3261G Medical Science I, Ann Arbor, MI 48109-5602, USA; ^10^Cancer Research and Biostatistics, 1730 Minor Ave, Suite 1900, Seattle, WA 98101, USA; ^11^MD Anderson Cancer Center, Sarcoma Medical Oncology, 1400 Holcombe Blvd, Unit 450, Houston, TX 77030, USA

In the article titled “SARC018_SPORE02: Phase II Study of Mocetinostat Administered with Gemcitabine for Patients with Metastatic Leiomyosarcoma with Progression or Relapse following Prior Treatment with Gemcitabine-Containing Therapy” [1], the second author, Dr. Ballman, was paid by Lilly and only recently realized that gemcitabine is a Lilly product and apologizes for not declaring this sooner.

## References

[1] Choy E., Ballman K., Chen J. (2018). SARC018_SPORE02: phase II study of mocetinostat administered with gemcitabine for patients with metastatic leiomyosarcoma with progression or relapse following prior treatment with gemcitabine-containing therapy. *Sarcoma*.

